# Prognostic value and clinicopathologic characteristics of L1 cell adhesion molecule (L1CAM) in a large series of vulvar squamous cell carcinomas

**DOI:** 10.18632/oncotarget.8353

**Published:** 2016-03-25

**Authors:** Marjolijn D. Trietsch, Maaike H.M. Oonk, Lukas J.A.C. Hawinkels, Rosalie Bor, Jaap D.H. van Eendenburg, Zina Ivanova, Alexander A.W. Peters, Hans W. Nijman, Katja N. Gaarenstroom, Tjalling Bosse

**Affiliations:** ^1^ Department of Pathology, Leiden University Medical Center, 2300 RC Leiden, The Netherlands; ^2^ Department of Gynaecology, University Medical Center Groningen, 9700 RB Groningen, The Netherlands; ^3^ Gastroenterology-Hepatology and Molecular Cell Biology, Leiden University Medical Center, 2300 RC Leiden, The Netherlands; ^4^ Institute for Pathology and Parasitology, Bulgarian Academy of Sciences, 1040 Sofia, Bulgaria; ^5^ Department of Gynaecology, Leiden University Medical Center, 2300 RC Leiden, The Netherlands

**Keywords:** L1 cell adhesion molecule, L1CAM, vulvar cancer, squamous cell carcinoma, survival

## Abstract

**Background:**

Vulvar cancer treatment is mostly curative, but also has high morbidity rates. In a search for markers that can identify patients at risk of metastases, we investigated the prognostic value of L1-cell adhesion molecule (L1CAM) in large series of vulvar squamous cell carcinomas (VSCCs). L1CAM promotes cell motility and is an emerging prognostic factor for metastasis in many cancer subtypes.

**Results:**

L1CAM expression was observed at the invasive front or in spray-patterned parts of 17% of the tumours. L1CAM-positive tumours expressed vimentin more often, but L1CAM expression was not associated with *TP53* or *CTNNB1* mutations. Five-year survival was worse for patients with L1CAM expression (overall survival 46.1% vs 63.6%, *P*=.014, disease specific survival 63.8% vs 80.0%, *P*=.018). Multivariate analysis indicates L1CAM expression as an independent prognostic marker (HR 2.9, 95% CI 1.10–7.68). An *in vitro* spheroid invasion assay showed decreased invasion of L1CAM-expressing VSCC spindle cells after treatment with L1CAM-neutralising antibodies.

**Materials and Methods:**

Paraffin-embedded tumour tissue from two cohorts (N=103 and 245) of primary VSCCs were stained for L1CAM, vimentin and E-cadherin. Patients of the first cohort were tested for human papilloma virus infection and sequenced for *TP53* and *CTNNB1* (β-catenin) mutations. The expression of L1CAM was correlated to clinical characteristics and patient survival.

**Conclusion:**

This is the first study to show high L1CAM-expression at the infiltrating margin of VSCC's. L1CAM-expressing VSCCs had a significantly worse prognosis compared to L1CAM-negative tumours. The highest expression was observed in spindle-shaped cells, where it might be correlated to their invasive capacity.

## INTRODUCTION

Vulvar cancer is the fourth most common gynaecological malignancy affecting approximately 2 in 100.000 women each year in developed countries [1, 2]. Vulvar cancer typically occurs in postmenopausal women: the mean age of diagnosis is 70 years [3, 4]. Two subgroups of vulvar squamous cell carcinoma (VSCC) are currently recognised. The first generally affects younger patients and is associated with infection by the human papilloma virus (HPV). The second develops independently from HPV infection and is associated with mutations in the *TP53* gene [5].

Patients diagnosed with vulvar cancer at an early stage generally have a good prognosis (90% 5-year survival for FIGO stage 1 patients) [3]. However, some patients suffer from rapidly progressing tumours that often recur and metastasize. Surgical treatment of early stage vulvar cancer is curative in most cases, but unfortunately, it also results in high morbidity rates [6, 7]. Researchers have tried to find prognostic markers that can differentiate patients who require aggressive (surgical) treatment from patients who would benefit from a more conservative and less invasive approach. This can include less radical surgical margins or to waive lymph node dissection or sentinel node procedure [8]. Despite these efforts, no prognostic markers are currently used in the clinical management of VSCC patients, except for lymph node metastasis, which is currently considered the most accurate predictor for prognosis [8, 9].

L1 cell adhesion molecule (L1CAM, or CD171) is thought to be one of the many factors involved in the induction of Epithelial-to-Mesenchymal Transition (EMT), responsible for the gain of invasive properties of cancer cells. L1CAM is a membrane glycoprotein that plays a crucial role in neural development where it has a dual mechanism: it can either stimulate cell adhesion, or it can promote cell motility. In normal adult tissue, L1CAM is only expressed by nerve tissue, leukocytes and renal tubules of the kidney, whereas in cancer it has also been reported to be expressed on tumour cell surface [10–12]. In tumour cells, L1CAM can switch from a cell adhesion to a cell motility promoting role, which is demonstrated by its stimulating effect on invasive growth of tumour cells [12, 13]. This is also illustrated by studies showing high L1CAM expression (sometimes even exclusively), at the invasive border of tumours [13, 14]. Finally, L1CAM can induce a more invasive phenotype in cell lines [15].

The prognostic significance of L1CAM expression has been addressed in many different types of cancer, including gynaecological cancers [14, 16–23]. Recently, two large studies showed the prognostic significance of L1CAM in low grade endometrioid endometrial cancers [24, 25]. L1CAM was found to be expressed in invasive areas of epithelial ovarian cancer and was correlated with poor clinical outcome and unfavourable clinicopathological features of the disease [21].

There are several hypotheses regarding the underlying mechanism of L1CAM upregulation in cancer. The three dominant hypotheses are that L1CAM is upregulated by mutant p53 [26], through Wnt-signalling [14, 26] or through the induction of TGF-β family members [26, 27]. L1CAM expression has not been examined in vulvar cancer before, but some studies have reported a relation between morphological features of EMT and a worse survival in vulvar cancer [28, 29].

In this study, we investigated the expression of L1CAM in a large series of 348 VSCC patients from two different academic hospitals and correlated it with survival. In order to further understand the process of L1CAM upregulation, clinicopathological characteristics and markers for EMT were studied in one of the cohorts. Finally, in a pilot in vitro study we have examined the role of L1CAM in invasion of vulvar cancer cells.

## RESULTS

From the Leiden cohort, 103 patients were included, and tumour sections from all patients were analysed for L1CAM. The average age at diagnosis was 71 years and the mean follow-up time was 4 years. Table 1 lists the characteristics of all included patients for the study cohort.

**Table 1:** Patients characteristics of the leiden cohort (n=103)

Characteristic			Value	
Follow up	– mo	(SD)	48.7	(36.1)
Age at diagnosis	– year	(SD)	70.7	(13.6)
Duration of symptoms	– mo	(IQR)	5.0	(2.0-17.3)
FIGO stage	– n	(%)		
Stage 1			27	(26)
Stage 2			36	(35)
Stage 3			29	(28)
Stage 4			11	(11)
Lymph node metastases	– n	(%)	39	(38)
Extracapsular growth	– n	(%)	17	(17)
Tumor size	– mm	(SD)	31.8	(21.7)
Infiltration depth	– mm	(IQR)	6.0	(4.0-10.0)
Positive resection margins	– n	(%)	21	(20.4)
Disease status	– n	(%)		
Complete remission			80	(78)
Local recurrence			20	(19)
Regional recurrence			9	(9)
Died			56	(54)
Disease specific death			25	(24)
5-yr overall survival	– %	(SD)	52.5	(5.1)
5-yr disease specific survival	– %	(SD)	74.7	(4.5)
5-yr disease free survival	– %	(SD)	30.0	(5.0)

**Figure 1:**
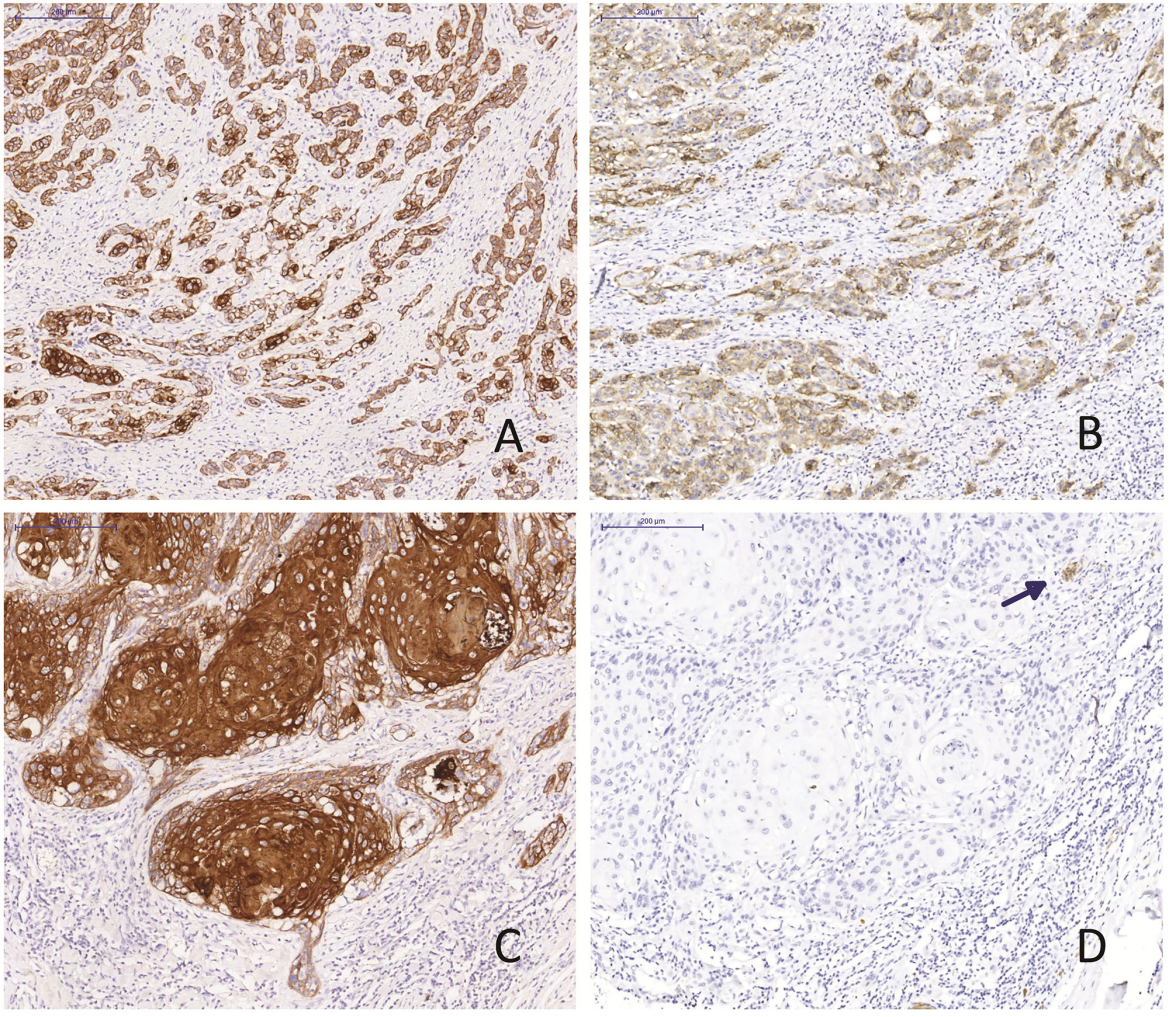
L1CAM expression Two vulvar squamous cell carcinomas with spindle cell morphology (**A** and **B**) and solid growth pattern (**C** and **D**) stained for keratin (**A** and **C**) and L1CAM (**B** and **D**). Arrowhead: nerve axon as an internal positive control.

Of the 103 patients in the study cohort, 16 (16%) were positive for L1CAM (Table 2). Figure 1 shows an example of an L1CAM positive tumour. All moderate to strong expressing cells were found at the invasive border of the tumours or areas with pronounced spindle-cell morphology (Figure 1). None of the more differentiated or solid, keratinizing tumours showed any L1CAM positivity. HPV was detected in 17 out of the total 103 patients (17%) and *TP53* mutations in 56 patients (54%). Previous research from our group has shown that VSCC with spindle cell morphology were more likely to carry *TP53* mutations and that spindle cell morphology was exclusively found in HPV negative patients [29]. Although L1CAM expression was more frequently seen in spindle patterned tumours, there was no relation between L1CAM upregulation and *TP53* mutations and/or HPV infection. No *CTNNB1* mutations or aberrant nuclear β-catenin expression were detected in any of the samples. L1CAM upregulation was not associated with changes in e-cadherin expression, since all tumours express e-cadherin. Vimentin expression in the tumour was detected in 29 samples (28.2%) and was correlated to L1CAM expression (Spearman's rho 0.349, *P*=0.001) (Table 3). An example of vimentin and L1CAM expression at the invasive border of a tumour is shown in Supplementary Figure S3.

**Table 2:** Comparison of patient characteristics for L1CAM positive and negative tumours in the leiden cohort

Outcome			L1CAM positive		L1CAM negative		p-value
			n=16	(16%)	n=87	(84%)	
Follow up	– mo	(SD)	29.0	(34.5)	52.3	(35.4)	0.017*
Age at diagnosis	– yr	(SD)	70.5	(13.1)	70.8	(13.8)	0.939
Duration of symptoms	– mo	(IQR)	4.0	(2.3 - 73.5)	5.0	(2.0 - 14.8)	0.573
FIGO stage	– n	(%)					0.023*
stage 1			2	(13)	25	(29)	0.227
stage 2			6	(38)	30	(35)	0.784
stage 3			3	(19)	26	(30)	0.547
stage 4			5	(31)	6	(7)	0.013*
Lymph node metastases	– n	(%)	8	(50)	31	(36)	0.401
Extracapsular growth			6	(38)	11	(13)	0.024*
Tumor size	– mm	(SD)	39.5	(19.2)	30.6	(21.9)	0.156
Infiltration depth	– mm	(IQR)	8.0	(5.5 - 13.3)	6.0	(3.5 - 9.0)	0.145
Positive resection margins	– n	(%)	7	(43.8)	14	(16.1)	0.019*
Disease status	– n	(%)					
Complete remission			9	(56)	71	(82)	0.045*
Local recurrence			2	(13)	18	(21)	0.771
Regional recurrence			0	(0)	9	(10)	
Died			13	(81)	43	(49)	0.027*
Disease specific death			7	(64)	18	(24)	0.012*
5-yr Overall survival	– %	(SD)	18.8	(10)	58.7	(6)	0.001*
5-yr Disease specific survival	– %	(SD)	42.8	(15)	79.3	(5)	0.013*
5-yr Disease free survival	– %	(SD)	30.0	(15)	41.6	(7)	0.266

**Table 3:** Correlating molecular markers to L1CAM upregulation in the leiden cohort

Outcome			L1CAM positive		L1CAM negative		χ^2^ p-value	Spearman correlation	p-value
			n=16	(16%)	n=87	(84%)			
HPV positive	– n	(%)	1	(12)	16	(18)	0.462	−0.118	0.233
*TP53* mutation	– n	(%)	10	(63)	46	(53)	0.278	0.070	0.482
HPV and/or *TP53*	– n	(%)	11	(69)	59	(68)	0.595	0.007	0.942
Vimentin	– n	(%)	10	(67)	17	(24)	0.004	0.349	0.001*

### Comparison of survival data

**Figure 2:**
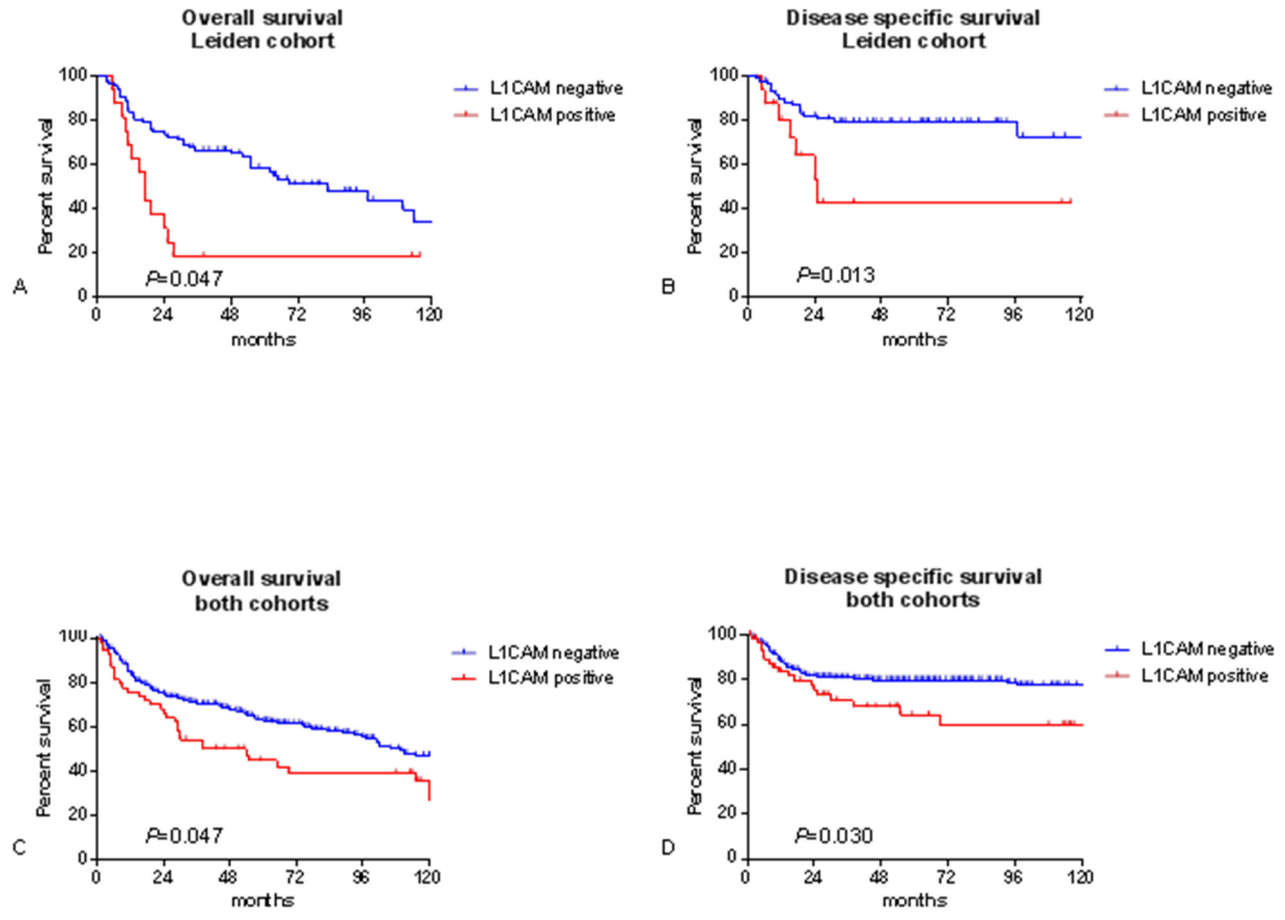
Survival curves Kaplan meier survival curves for the leiden cohort, n=103 **A, B.** and both cohorts combined, n=348 **C, D.** P-values for log-rank test.

Clinical data from the Leiden cohort were compared for patients with and without L1CAM positive tumours and listed in Table 2. Patients with L1CAM positive tumours presented more often at the highest FIGO stage (31.1% vs 6.9%, *P*=.023) and if they had lymph node metastasis, it was more likely to be bilateral (31.3% vs 8.0%, *P*=.029) and with extracapsular growth (37.5% vs 12.6%, *P*=.024). Patients without L1CAM staining were more likely to reach complete remission (81.6% vs 56.2%, *P*=.045). L1CAM positive patients had a worse 5-year overall and disease specific survival (18.8% vs 58.7%, log rank *P*=.001 and 42.8% vs 79.3%, log rank *P*=.013, respectively) (Figure 2).

Patients with L1CAM-expressing tumours had a 2.9 times higher risk of dying from their cancer than patients lacking L1CAM expression (HR 2.9, 95% CI 1.10 – 7.68) when corrected for the possible confounders lymph node metastasis, tumour size and *TP53* mutations. Also for overall survival, L1CAM remained to be an independent prognostic factor when corrected for these confounders (HR 2.28, 95% CI 1.13 – 4.57) (Table 4).

**Table 4:** Multivariate cox regression analysis for the leiden cohort (n=103)

Disease specific survival		
Variable	HR	95% CI
Lymph node metastasis	5.58	2.29 - 13.62
Tumour size (mm)	1.02	1.00 - 1.04
*TP53* mutation	2.48	0.98 - 6.24
L1CAM staining	2.91	1.10 - 7.68
**Overall survival**		
**variable**	**HR**	**95% CI**
Lymph node metastasis	3.33	1.91 - 5.78
Tumour size (mm)	1.03	1.02 - 1.04
*TP53* mutation	1.50	0.84 - 2.67
L1CAM staining	2.28	1.13 - 4.57

### Validating prognostic data in an independent cohort

Of the 245 patients in the independent TMA cohort, the average age at diagnosis was 72 years and the mean follow-up time was 5 years. Table 5 lists the characteristics of all included patients in the TMA cohort.

**Table 5:** Patients characteristics of the TMA cohort (n=245)

Characteristic	n=245		Value	
Follow up	– mo	(SD)	60.0	(50.5)
Age at diagnosis	– year	(SD)	71.7	(13.1)
FIGO stage	– n	(%)		
Stage 1			51	(21)
Stage 2			94	(38)
Stage 3			67	(27)
Stage 4			33	(13)
Lymph node metastases	– n	(%)	92	(43)
Extracapsular growth	– n	(%)	40	(16)
Tumor size	– mm	(SD)	33.0	(17.2)
Infiltration depth	– mm	(IQR)	7.0	(3.8-10.0)
Positive resection margins	– n(%)		21	(8.6)
Disease status	– n	(%)		
Complete remission			154	(63)
Local recurrence			50	(20)
Regional recurrence			14	(6)
Distant recurrence			6	(2)
Died			120	(49)
Disease specific death			50	(20)
5-yr Overall survival	– %	(SD)	59.6	(3.0)
5-yr Disease specific survival	– %	(SD)	78.2	(2.6)
5-yr Disease free survival	– %	(SD)	64.5	(3.0)

Of these 245 patients, 44 (18%) overexpressed L1CAM (Table 6). The five year overall and disease specific survival was worse for patients with L1CAM expression, but it did not reach statistical significance (49.4% vs 61.3%, log rank *P*=0.074 and 70% vs 80%, log rank *P*= 0.159) (Table 6). Since no material was available for mutation and HPV analysis, Cox regression analysis was performed correcting for lymph node metastasis and tumour size only. Patients with L1CAM positive tumours showed a trend towards increased risk of dying, with a hazard ratio of 1.58 (95% CI 0.79 – 3.19) for disease specific survival and 1.48 (95% CI 0.93 – 2.35) for overall survival, these hazard ratios however did not reach statistical significance (Supplementary Table S1).

**Table 6:** Comparison of patient characteristics for L1CAM positive and negative tumours in the TMA cohort

Characteristic			L1CAM positive		L1CAM negative		p-value
			n=44	(18%)	n=201	(82%)	
Follow up	– mo	(SD)	55.2	(50.2)	61.9	(50.9)	0.432
Age at diagnosis	– yr	(SD)	73.1	(13.0)	71.2	(13.0)	0.381
FIGO stage	– n	(%)					0.031*
stage 1			7	(16)	44	(22)	
stage 2			13	(30)	81	(40	
stage 3			20	(46)	47	(23)	
stage 4			4	(9)	29	(14)	
Lymph node metastases	– n	(%)	21	(48)	71	(35)	0.104
Extracapsular growth			9	(21)	31	(15)	0.219
Tumor size	– mm	(SD)	35.8	(22.4)	32.8	(16.3)	0.303
Infiltration depth	– mm	(IQR)	7.0	(4.0-10.0)	7.0	(3.5-10.2)	0.912
Lymfangio invasion	– n	(%)	11	(25)	28	(14)	0.112
Positive resection margins	– n	(%)	5	(11)	16	(8)	0.386
Disease status	– n	(%)					
Complete remission			22	(50)	132	(66)	0.059
Local recurrence			9	(21)	36	(18)	0.413
Regional recurrence			2	(5)	10	(5)	
Distant recurrence			3	(7)	3	(1)	
Died			27	(61)	93	(46)	0.095
Disease specific death			12	(27)	38	(19)	0.219
5-yr Overall survival	– %	(SD)	49.4	(8.2)	61.3	(3.7)	0.074
5-yr Disease specific survival	– %	(SD)	70.4	(8.2)	80.3	(3.0)	0.159
5-yr Disease free survival	– %	(SD)	57.5	(9.4)	71.0	(3.6)	0.188

When taking all survival data together, thus creating a combined cohort of 348 patients (Supplementary Table S2) of which 60 (17%) were positive for L1CAM (Table 7). The 5 year overall and disease specific survival was significantly worse for patients with L1CAM positive tumours (46.1% vs 63.6%, log rank *P*=.014 and 63.8% vs 80.0%, log rank *P*=.018) (Table 7). Patients with L1CAM positive tumours were more likely to have lymph node metastasis than patients without L1CAM expression (48.3% vs 35.4%, *P*=.048) (Table 7).

**Table 7:** Comparison of patient characteristics for L1CAM positive and negative tumours in both the leiden and the TMA cohort

Characteristic			L1CAM positive		L1CAM negative		p-value
			n=60	(17%)	n=288	(83%)	
Follow up	– mo	(SD)	48.2	(47.7)	59.0	(46.9)	0.108
Age at diagnosis	– yr	(SD)	72.8	(13.0)	70.7	(13.3)	0.258
FIGO stage	– n	(%)					0.124
stage 1			9	(15)	69	(24)	
stage 2			19	(32)	111	(39)	
stage 3			23	(38)	73	(25)	
stage 4			9	(15)	35	(12)	
Lymph node metastases	– n	(%)	29	(48)	102	(35)	0.048*
Extracapsular growth			15	(25)	42	(15)	0.101
Tumor size	– mm	(SD)	36.7	(21.6)	32.1	(18.8)	0.092
Infiltration depth	– mm	(IQR)	7.0	(4.4 - 11.0)	6.5	(3.5 - 10.0)	0.478
Positive resection margins			11	(18.3)	31	(10.8)	0.122
Disease status	– n	(%)					
Complete remission			31	(52)	203	(71)	0.006*
Local recurrence			13	(2)	58	(20)	
Regional recurrence			2	(3)	18	(6)	
Distant recurrence			4	(7)	10	(4)	
Died			40	(67)	136	(47)	0.007*
Disease specific death			19	(32)	56	(19)	0.023*
5-yr Overall survival	– %	(SD)	46.1	(7.2)	63.6	(3.0)	0.014*
5-yr Disease specific survival	– %	(SD)	63.8	(7.4)	80.0	(2.5)	0.018*
5-yr Disease free survival	– %	(SD)	57.5	(9.4)	71.0	(3.6)	0.188

The multivariate Cox regression analysis for both cohorts combined, correcting for lymph node metastasis and tumour size provided a hazard ratio of 1.58 (95% CI 1.08 – 2.32) for overall survival and 1.70 (95% CI 0.97 – 2.97) for disease specific survival (Table 8).

**Table 8:** Multivariate cox regression analysis for both the leiden and the TMA cohort (n=348)

Disease specific survival		
Variable	HR	95% CI
Lymph node metastasis	6.1	3.35 - 11.10
Tumour size (mm)	1.02	1.01 - 1.03
L1CAM staining	1.70	0.97 - 2.97
**Overall survival**		
**variable**	**HR**	**95% CI**
Lymph node metastasis	2.13	1.54 - 2.93
Tumour size (mm)	1.02	1.02 - 1.03
L1CAM staining	1.58	1.08 - 2.32

### L1CAM inhibition decreases invasion of VSCC spindle shaped but not cobble shaped cells

**Figure 3:**
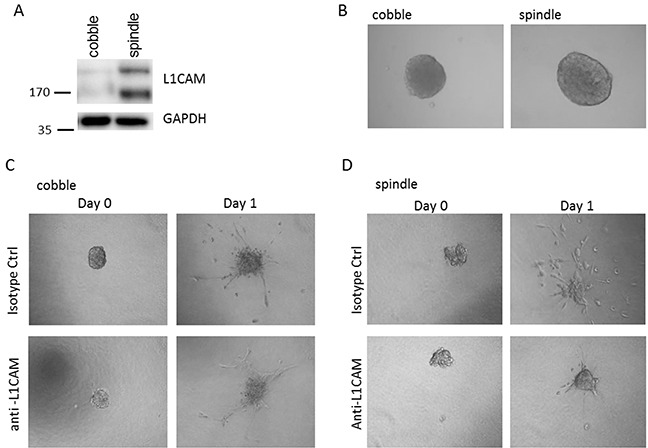
Spheroid invasion assay **A.** Spindle shaped cells highly express L1CAM, while L1CAM expression on cobble shaped cells is very low. **B.** Spindle and cobble cells form spheroids equally well and invade when embedded in a collagen matrix (**C.** and **D.**). Note that the invasion of the non-L1CAM expressing cobble shaped cells (C) is hardly affected by L1CAM neutralising antibodies, while spindle cell invasion can be strongly inhibited by the L1CAM neutralising antibodies (D)

To analyse the potential role for L1CAM in tumour cell invasion, we isolated spindle- and cobble-shaped cells from one VSCC which contained both components. Epithelial origin of the spindle- and cobble cells was confirmed by positive immunohistochemical stainings for pan-cytokeratin and presence of identical *TP53* mutations detected in the tumour from which the cells were derived. L1CAM expression was analysed by western blot. Figure 3A shows that spindle shaped cells highly express L1CAM, whereas L1CAM expression on cobble shaped cells is very low. Next, both cell types were grown as spheroids and embedded in collagen type-I matrix to study the invasive properties (Figure 3B). In the presence of an L1CAM neutralising antibody the invasion of the spindle cell population can be strongly inhibited, while the invasion of the non-L1CAM expressing cobble shaped cells is hardly affected (Figure 3C and 3D). This experiment stresses the importance of L1CAM for the invasive potential of the spindle cells and opens possibilities to explore these antibodies in a therapeutic setting.

## DISCUSSION

L1CAM expression has shown to be a marker for poor disease outcome in several types of cancer [14, 16–23] and this is study is the first to evaluate the prognostic capacity in vulvar cancer. Taking both the Leiden and the TMA cohort together, L1CAM was upregulated in 60 (17%) of all included VSCC. Patients with L1CAM expressing tumours have a significantly worse 5 year disease specific survival compared to patients with normal L1CAM expression (80% vs 64%). Furthermore our pilot in vitro data show an important role specifically for L1CAM in the invasive properties of spindle shaped vulvar cancer cells.

There are three hypotheses how L1CAM is upregulated in cancer. Studies in colorectal cancer cell lines have shown that β-catenin (*CTNNB1*) mutations, and the subsequent aberrant activation of the Wnt signalling pathway, result in upregulation of L1CAM [14]. Despite these findings in colorectal cancer, our work and that of others on vulvar cancers shows no evidence of *CTNNB1* gene mutations, nor nuclear β-catenin expression [28, 33]. Therefore, we do not expect that Wnt-signalling through β-catenin is a major factor in the upregulation of L1CAM in vulvar cancer.

P53 has also been postulated as a regulator of L1CAM expression and *TP53* is frequently mutated in vulvar cancer [26]. In our current study, there was no correlation between *TP53* mutations and L1CAM positivity. Since HPV infection can alter the function of wild type p53, a relation between L1CAM expression and p53 aberration by *either* HPV infection or *TP53* mutation was investigated, but we did not find any correlation. We therefore concluded that p53 might not be driving L1CAM expression in vulvar cancer. A third alternative is that L1CAM expression is upregulated in the process of EMT [41, 42]. EMT-like changes have been described in vulvar cancer by us [29] and others [28] and was associated with poor clinical outcome. Our current findings show that L1CAM expression is exclusively found in areas with EMT-like growth (at the invasive border), or in tumours with predominant spray patterned growth, suggesting an association between EMT and L1CAM. Moreover, we confirmed that spindle shaped epithelial tumour cells highly express L1CAM protein, in contrast to their cobble shaped counterparts, which show very low or absent L1CAM.

Together, our findings indicate that L1CAM upregulation may be a consequence of EMT-like changes in vulvar cancer. Our spheroid invasion assay suggests that the invasion of these spindle shaped vulvar tumour cells can be strongly inhibited by treatment with L1CAM neutralising antibodies. These findings are in line with other tumour models, where treatment with L1CAM neutralising antibodies seems to inhibit tumour growth and metastasis [37]. Therefore our work contributes to the growing evidence showing efficacy of inhibiting tumour growth by L1CAM antibodies and stresses the need for further evaluating its therapeutic potential in vulvar and other cancers.

The strength of this study is the relatively large number of included patients with vulvar cancer. When combining the two cohorts, the statistical power of our analyses increases, which underlines the potential prognostic value of L1CAM expression in vulvar cancer. We do want to encourage other institutes to reproduce our study in order to validate our hypothesis. Because of the low incidence of vulvar cancer, we suggest these should be multicentre studies containing at least hundreds of patients.

A potential weakness of this study is its retrospective design. Since therapies change and improve over time, using samples dating back to 1984 might distort the survival analysis. Also, by excluding patients who underwent small excision biopsies and needed no further surgical excision, small sized tumours that have a relatively good prognosis might have been excluded. On the other hand, patients that did not receive surgical treatment were also excluded because of the large size or metastasis of their tumour. By including tumour size in the multivariate analysis, we have corrected for this possible bias. For the TMA cohort, only tissue micro arrays were available. Although three cores from different locations in the tumour were included for each patient, chances still are that L1CAM positive areas of the tumour were missed when taking these tissue cores. While the percentage of L1CAM positive samples was comparable between the study and the TMA cohort (16 and 18%), patients might have been allocated to the L1CAM negative group, thus diluting the effect L1CAM expression has on prognosis. The detected differences in survival are therefore on the safe side and might in fact be even more significant if full slides would have been available for both cohorts in this study.

In summary, we have shown for the first time that L1CAM is expressed in 17% of the VSCC's and that it is an independent prognostic factor for both overall and disease specific survival. Therefore L1CAM proves to have potential as a reliable biomarker that can be used to discriminate high risk from low risk vulvar cancer patients. We have studied and validated this prognostic significance in a large cohort of a relatively rare cancer type. Our results implicate that, unlike in other cancers, p53 and Wnt-signalling do not appear to play a dominant role in the (up)regulation of L1CAM. More likely, our data point towards a link between EMT and L1CAM expression in VSCC. We can conclude that L1CAM expression represents a promising prognostic biomarker in vulvar cancer. In addition, the potential to use L1CAM as a target for therapy based on our vulvar cancer cell invasion assays, warrants further investigation.

## MATERIALS AND METHODS

### Patient selection and sample collection

All patients samples were handled according to the medical ethical guidelines described in the Code of Conduct for Proper Secondary Use of Human Tissue of the Dutch Federation of Biomedical Scientific Societies. (www.federa.org, an English translation of the Code can be found here: http://www.federa.org/sites/default/files/digital_version_first_part_code_of_conduct_in_uk_2011_12092012.pdf)

Two cohorts of patients from two different referral cancer centres were included in this study. The first cohort exists of 108 patients with primary vulvar squamous cell carcinoma who were surgically treated at the Leiden University Medical Center between 2000 and 2009. Of these patients, 5 tissue blocks did not contain sufficient tumour tissue anymore and were excluded, therefore resulting in a cohort of 103 samples. Patients were also excluded if they had received chemotherapy or radiotherapy in the pelvic area prior to the operation, or if they had received systemic immunosuppressive therapy (n=9). Patients who underwent excision biopsies without further surgery were excluded, because biopsies do not contain enough tumour material (n=11). Clinical and follow-up data were retrospectively retrieved from patient medical records and the institutional cancer registration database. Follow up ended in December 2012.

The second cohort consists of 298 patients with primary vulvar squamous cell carcinoma who were surgically treated at the University Medical Center Groningen between 1984 and 2001. Since 1984, clinicopathological and follow-up data of all patients referred to the Department of Gynecologic Oncology of the University Medical Center Groningen, the Netherlands are prospectively collected during standard treatment and follow-up. All consecutive vulvar squamous cell cancer patients with T1-2 tumors were selected. Twenty-five patients were excluded because they did not undergo inuinofemoral lymphadenectomy, which was a selection criterium in the original study [30]. In 18 cases this was because of general bad health, in the other 7 cases because of FIGO stage 1A disease. With the tissue samples of these patients, a tissue micro array (TMA) was built as previously described [30]. Therefore, this cohort will be referred to as the ‘TMA cohort’. Patients were excluded when they had been treated with preoperative radiotherapy. Fiftythree patients were removed from analysis because too many cores were missing from the TMA, resulting in a cohort of 245 patients in total.

Tumour staging for both cohorts was performed according to the FIGO system; the 1995 staging instead of the revised 2009 staging was used because of the retrospective design of the study [31, 32]. In the multivariate survival analysis, we corrected for lymph node metastasis and tumour size instead of FIGO stage, because these factors are not subject to revisions of staging systems over time. These cohorts have been described before [29, 30, 33].

### Immunohistochemistry

Formalin fixed, paraffin embedded (FFPE) tissue blocks were collected for all included patients. 4μm sections were cut and sections were stained with haematoxylin and eosin to select representative tumour containing tissue blocks and areas.

The selected sections were stained using anti L1CAM antibody clone 14.1, 1:500 (Covance, Princeton, NJ, USA) and counterstained with haematoxylin as described before [34]. Stained sections were analysed by one PhD-candidate and one gynaecopathologist (MDT and TB) separately, blinded for patient characteristics and outcome data. L1CAM expression was marked “positive” if 5% or more of the tumour cells stained moderate or strong for L1CAM. All other staining patterns were grouped “L1CAM negative”, which included 1) completely negative tumours with positive internal control, 2) tumours with scattered positive tumour cells (<5%) or 3) very weak stained tumours (intensity was compared with internal control).

L1CAM staining of nerve axons was used as an internal positive control. A consensus was reached for all samples.

The Leiden cohort was also stained for β-catenin antibody clone 14 1:800, e-cadherin C20820 1:100 (both BD Biosciences, Franklin Lakes, NJ, USA) and vimentin V9-2B 1:50 (Department of Pathology, Leiden University Medical Center, Leiden, the Netherlands) according to the manufacturers protocol.

The TMA cohort consisted of 4 Tissue Micro Arrays (TMA) with tumour samples of 298 squamous cell vulvar cancer patients [30]. For the TMA cohort, 4μm sections of the TMA were cut. Samples on the TMA were scored for each tissue core separately, later combining the results from the three cores per patient. A patient with at least one core with moderate to strong L1CAM expression was scored positive for L1CAM, and negative for L1CAM if at least two cores were present and all negative. If two or more cores were missing or missing for more than 50%, and the remaining core was not scored as positive, the patient was marked as missing and removed from further analysis.

### Tumour cell isolation and spheroid invasion assay

From fresh residual tumour tissue (81 year old patient, FIGO stage IVa) collected after diagnostic use, tumour cells were isolated. Keratin staining of the tumour showed both solid and spindle shaped tumour cells (Supplementary Figure S1). The tumour was tested negative for HPV as described before [29]. Cells were harvested after overnight incubation at room temperature in 5 ml DMEM (Invitrogen, United Kingdom) containing 1 mg/ml collagenase and 1 mg/ml dispase. Next day, cells were washed and subsequently incubated in RPMI 1640 containing 10% Fetal Calf Serum (F7524, Sigma-Aldrich, USA) 50 U penicillin per ml and 50 μg streptomycin per ml (G1397, Sigma-Aldrich, USA. During cell culture cobble shaped and spindle shaped tumour cells were identified and were separated. Both cell types were cultured further until pure populations were obtained. Supplementary Figure S2 show the morphologic features of the cells. To characterise the cells *TP53* Sanger sequencing was performed, showing a *TP53* R248Q mutation identical to the original tumour both in the spindle shaped and the cobble shaped cell population, reaffirming the epithelial origin of both cell types. L1CAM expression on both cell types was evaluated by western blot analysis as described before [35] using mouse anti- L1CAM antibodies (clone L1-9.3/2a, 2.3 μg/ml in TBST) and chemoluminescent detection.

The spheroid invasion assay was performed as described before [36]. In short, spindle and cobble VSSCs were grown to spheroids (500 cells per spheroid) by plating them on agarose-coated 96 well plates. After 48h spheroids were harvested and embedded in a collagen type-I matrix in the presence of 40 μg/ml isotype control or L1CAM neutralising antibodies (clone L1-9.3), both kindly provided by Prof. Dr. Altevogt [37]. Invasion of the cells into the collagen matrix was analysed by microscopy and pictures were taken at 1 day after embedding at 10x magnification (Olympus microscope). At least 10 spheroids per conditions were analysed and the experiment was repeated two times.

### HPV and mutation analysis

Mutation analysis and HPV typing were performed on the study cohort of 103 patients. The pancytokeratin-stained slides were used to select an area consisting of at least 70% tumour cells. Three 0.6-mm diameter tissue cores of variable length were taken from the selected area in the FFPE blocks. DNA isolation was performed in an automated fashion as described previously using the Tissue Preparation System (Siemens Healthcare Diagnostics, Malvern, Pennsylvania, USA) [38]. DNA quality was tested by multiplex quality PCR that amplified 150-, 255-, 343-, and 511-base pair products that were visualized using 2% agarose gel electrophoresis and scored for quality (scale, 0–4) (primer sequences available upon request).

The INNO-LiPA HPV Genotyping Extra Amp kit for in vitro diagnostic use (Innogenetics, Gent, Belgium), a highly sensitive hybridization assay, was used for HPV typing as described previously [39]. This assay can detect oncogenic and common HPV types.

For analysis of somatic mutations in the *TP53* gene, DNA sequencing was performed for exons 5–8 as described before [33]. Mutation genotyping of *CTNNB1* was performed using the GynCarta 2.0 panel [40], which covers 88% of the currently known *CTNNB1* mutations.

### Statistical analysis

Statistical analyses were conducted using the Predictive Analytics Software package (version 17, IBM-SPSS Statistics, Armonk, New York, USA). The independent *t*-test was used to compare baseline variables and Fisher's exact test to analyse categorical and normally distributed numerical data. The Shapiro–Wilk test was used to test for normality, and for data with a skewed distribution, the Mann–Whitney U test was used. Kaplan–Meier, the log-rank test, and Cox proportional hazard regression analysis were performed to analyse differences in survival between groups of patients with and without L1CAM expression. A *P* value of .05 was considered significant, corresponding to 95% confidence intervals (CIs). All tests were two-tailed. Results for normally distributed numerical data are presented as the mean with standard deviation (SD), and results for skewed numerical data are presented as the median with interquartile range (IQR).

## SUPPLEMENTARY FIGURES AND TABLES


