# Supplementary material for: Prognostic value and clinicopathologic characteristics of L1 cell adhesion molecule (L1CAM) in a large series of vulvar squamous cell carcinomas
Source: Oncotarget. 2016 Mar 25;7(18):26192–205. doi: 10.18632/oncotarget.8353 (PMC5041974; doi:10.18632/oncotarget.8353)
Supplement: Supplementary file 1 [file oncotarget-07-26192-s001.pdf]

SUPPLEMENTARY FIGURES AND TABLES

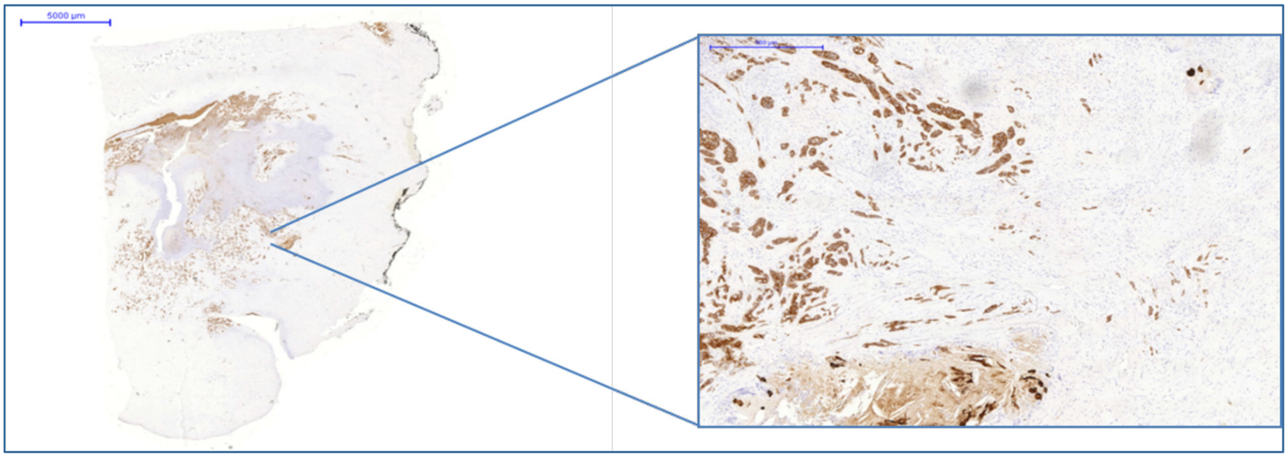

Supplementary Figure S1: Original tumour from which two cell line populations were isolated, stained for pancytokeratin showing both solid an spindle shaped tumour cells.

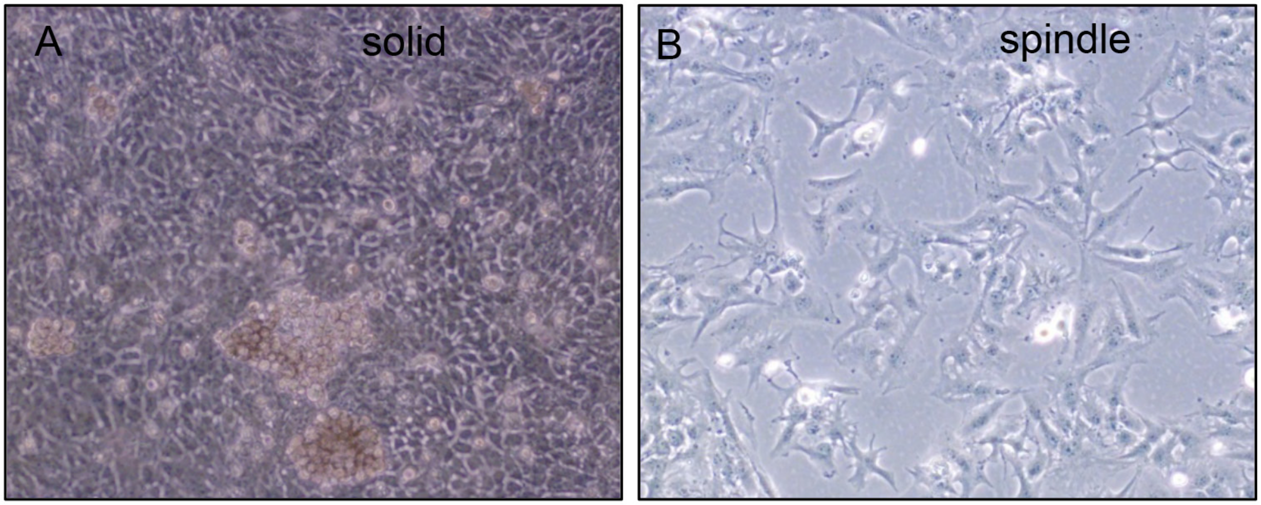

Supplementary Figure S2: Bright light microscopy images of the two cell populations consisting of cobble shaped cells from the 'solid' tumour **A.** and spindle shaped cells **B.**

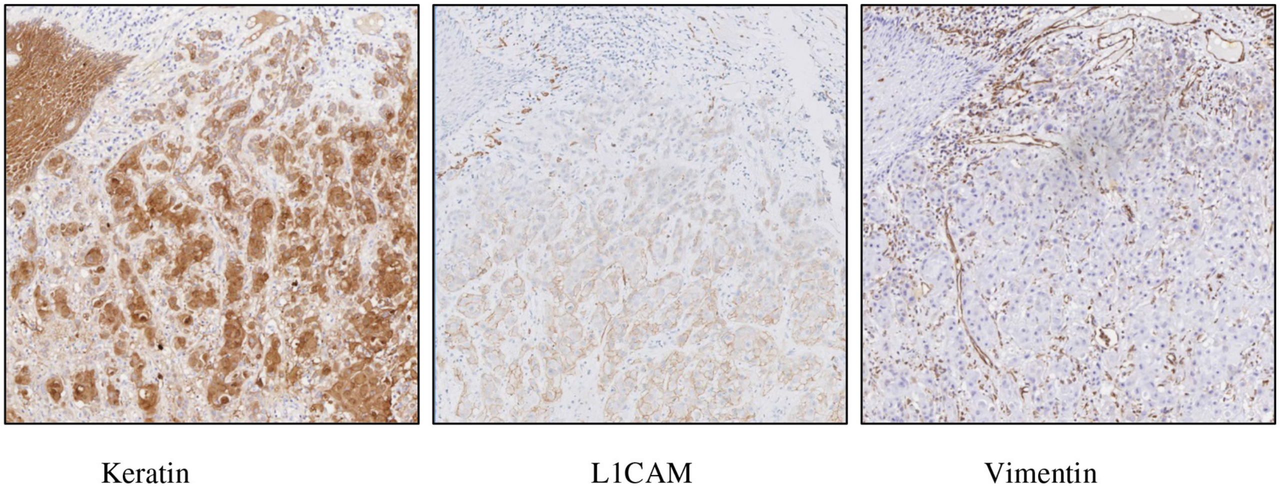

Supplementary Figure S3: Vimentin and Keratin expression in the tumour border of a vulvar cancer.

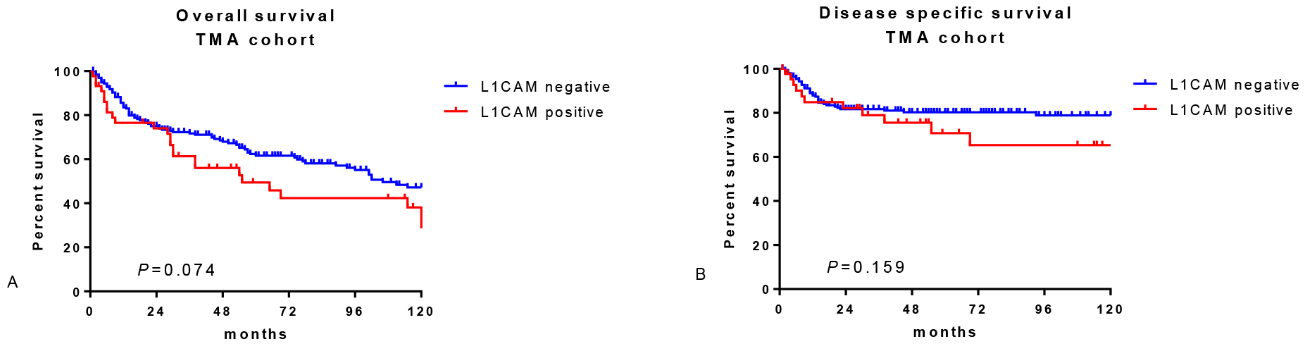

Supplementary Figure S4: Overall and disease specific survival curves of the TMA cohort (N=245).

Supplementary Table S1: Multivariate cox regression analysis for the TMA cohort (n=245)

Disease specific survival		
**variable**	**HR**	**95% CI**
Lymph node metastasis	7.26	3.17 - 16.62
Tumour size (mm)	1.02	1.01 - 1.04
L1CAM staining	1.58	0.79 - 3.19
**Overall survival**		
**variable**	**HR**	**95% CI**
Lymph node metastasis	1.91	1.28 - 2.85
Tumour size (mm)	1.02	1.01 - 1.03
L1CAM staining	1.48	0.93 - 2.35

Supplementary Table S2: Patients characteristics of both the leiden and the TMA cohort together (n=348)

Characteristic	n=348		Value	
Follow up	– mo	(SD)	57.1	(47.1)
Age at diagnosis	– year	(SD)	71.1	(13.2)
FIGO stage	– n	(%)		
Stage 1			78	(22)
Stage 2			130	(37)
Stage 3			96	(28)
Stage 4			44	(13)
Lymph node metastases	– n	(%)	131	(38)
Extracapsular growth	– n	(%)	57	(16)
Tumor size	– mm	(SD)	32.9	(18.8)
Infiltration depth	– mm	(IQR)	7.0	(4.0 - 10.0)
Positive resection margins	– n	(%)	42	(12.1)
Disease status	– n	(%)		
Complete remission			234	(67)
Local recurrence			71	(20)
Regional recurrence			20	(6)
Distant recurrence			14	(4)
Died			176	(51)
Disease specific death			75	(22)
5-yr Overall survival	– %	(SD)	60.4	(2.8)
5-yr Disease specific survival	– %	(SD)	77.5	(2.4)
5-yr Disease free survival	– %	(SD)	68.7	(3.4)
